# Computed Tomography Images of Fibrotic Pulmonary Sarcoidosis Leading to Chronic Respiratory Failure

**DOI:** 10.3390/jcm9010142

**Published:** 2020-01-05

**Authors:** Michiru Sawahata, Takeshi Johkoh, Takeshi Kawanobe, Chiyoko Kono, Yosikazu Nakamura, Masashi Bando, Koichi Hagiwara, Tamiko Takemura, Fumikazu Sakai, Noriharu Shijubo, Satoshi Konno, Tetsuo Yamaguchi

**Affiliations:** 1 Division of Pulmonary Medicine, Department of Medicine, Jichi Medical University, Shimotsuke 329-0498, Japan; bando034@jichi.ac.jp (M.B.); hagiwark@me.com (K.H.); 2 Department of Radiology, Kansai Rosai Hospital, Amagasaki 660-8511, Japan; johkoh-takeshi@kansaih.johas.go.jp; 3 Department of Respiratory Medicine, JR Tokyo General Hospital, Shibuya 151-8528, Japan; kawanobe-t@jreast.co.jp (T.K.); c-kouno@jreast.co.jp (C.K.); 4 Department of Public Health, Jichi Medical University, Shimotsuke 329-0498, Japan; nakamuyk@jichi.ac.jp; 5 Department of Pathology, Kanagawa Cardiovascular and Respiratory Center, Yokohama 236-0051, Japan; byori@med.jrc.or.jp; 6 Saitama Medical University International Medical Center, Department of Radiology, Hidaka 350-1298, Japan; 7 Department of Respiratory Medicine, JR Sapporo Hospital, Sapporo 060-0033, Japan; n-sijubo@jrhokkaido.co.jp; 8 Department of Respiratory Medicine, Faculty of Medicine and Graduate School of Medicine, Hokkaido University, Sapporo 060-0808, Japan; satkonno@pop.med.hokudai.ac.jp; 9 Department of Respiratory Medicine, Shinjuku Tsurukame Clinic, Shibuya 151-0053, Japan; yamatet@icloud.com

**Keywords:** sarcoidosis, fibrosis, upper lobe shrinkage, traction bronchiectasis, honeycomb lung, cyst, consolidation, chronic respiratory failure, home oxygen therapy, hilar mediastinal lymphadenopathy

## Abstract

Background: There is currently no consensus on the morphology of severe fibrotic pulmonary sarcoidosis, and we examined computed tomography (CT) findings and progression. Methods: We analyzed findings in 10 consecutive patients (three men, seven women) with pulmonary sarcoidosis requiring oxygen therapy for chronic respiratory failure, who were extracted from >2500 sarcoidosis patients (three hospitals, 2000–2018). Patients with comorbidities causing chronic respiratory failure were excluded. Results: Predominant findings were consolidations along the bronchovascular bundles comprising ‘central-peripheral band’, traction bronchiectasis, peripheral cysts/bullae, and upper lobe shrinkage. Traction bronchiectasis arose from opacities comprising ‘central-peripheral band’. Clustering of traction bronchiectasis at the distal side formed honeycomb lung-like structures in three patients. Upper lobe shrinkage progressed in seven patients together with progression of consolidations, ‘central-peripheral band’, traction bronchiectasis clusters, and cysts, while patients without shrinkage included two patients with severe multiple cysts without traction bronchiectasis. Restrictive ventilatory impairment developed in most patients. Pulmonary hypertension (PH) was detected radiologically in five patients, and chronic progressive pulmonary aspergillosis (CPPA) in four patients. Conclusions: During progression, consolidations comprising ‘central-peripheral band’ progressed together with traction bronchiectasis clusters and peripheral cysts, resulting in upper lobe shrinkage. This may lead to respiratory failure with possible complications such as PH and CPPA.

## 1. Introduction

Sarcoidosis is a granulomatous disease that causes different types of lesions in various organs throughout the body, most commonly in the respiratory organs. Causative antigens enter the airway and are thought to invade regional intrathoracic lymph nodes during the early stages of the disease, but the etiology remains unclear. Respiratory involvement is found in almost all patients with sarcoidosis, and varying types of pulmonary lesions and bilateral hilar-mediastinal lymphadenopathy (BHL) are observed; most patients enter remission and have good long-term outcomes, while up to 20% develop fibrotic lung disease [1]. Sarcoidosis-associated death occurs in 5–10% of patients, mainly from pulmonary fibrosis, as well as cardiac involvement and central nervous system involvement. However, there is currently no consensus on the morphology of severe fibrotic pulmonary sarcoidosis [1,2,3]. Given that this disease is relatively rare and decades of observation are required before development of pulmonary fibrosis can be seen, finding many patients with this condition is difficult. This is considered the main reason why fibrotic pulmonary sarcoidosis has not been well investigated.

Chest X-ray staging (Stage I, BHL only; Stage II, BHL plus pulmonary infiltration; Stage III, pulmonary infiltration only; Stage IV, pulmonary fibrosis) according to the criteria modified by Scadding in 1961 [4] is widely used to identify the locations of lesions in the clinical setting. Staging is associated with age [5], but the association with prognosis is insufficient. The mechanism underlying the distortion of lung architecture, which ultimately results in respiratory failure, is unknown. Considering that a clinical trial in patients with progressive fibrosing interstitial lung diseases with not only typical interstitial pneumonia but also other fibrotic patterns showed the efficacy of nintedanib [6], further understanding of proper indications in pulmonary sarcoidosis will require urgent clarification of detailed computed tomography (CT) findings of fibrotic pulmonary sarcoidosis.

Against this background, we extracted the most severe pulmonary sarcoidosis cases who developed chronic respiratory failure with oxygen therapy induction, examined their CT findings, and observed the progression of pulmonary fibrosis. We expected that this would provide insight into the development of chronic pulmonary sarcoidosis as well as the progression of pulmonary fibrosis and development of respiratory failure.

## 2. Methods

### 2.1. Patients

Ten consecutive pulmonary sarcoidosis patients (three men and seven women) requiring oxygen therapy for chronic respiratory failure were extracted from among more than 2500 sarcoidosis inpatients and outpatients at three institutions between January 2000 and September 2018. They were regarded as having fibrotic pulmonary sarcoidosis, and their CT findings were analyzed. Patients with other comorbidities (e.g., lethal pulmonary infection and heart failure) that could cause chronic respiratory failure were excluded. For example, at JR Sapporo Hospital, among 1112 sarcoidosis patients (432 men and 680 women), 13 patients required oxygen therapy. Of these 13 patients, we excluded five with complicating heart failure, three with lung cancer, one with eosinophilic pneumonia, and one without sufficient clinical information; eventually, three patients were included as study participants. 

Clinical information collected from the patients’ medical records included age at the time of sarcoidosis onset and oxygen therapy induction, gender, smoking history, and pulmonary or extrapulmonary manifestations of the disease. We obtained CT scans during inspiration with the patient lying supine. One respirologist and one radiologist examined the CT findings with a commercially available DICOM viewer. Routine laboratory tests and respiratory function tests, including vital capacity, forced expiatory volume in 1 s, and carbon monoxide diffusing capacity, had been performed during the clinical course.

This study was reviewed and approved by the Clinical Research Ethics Committee of Hokkaido University Hospital (017-0431).

### 2.2. CT Image Evaluation

**Figure 1:**
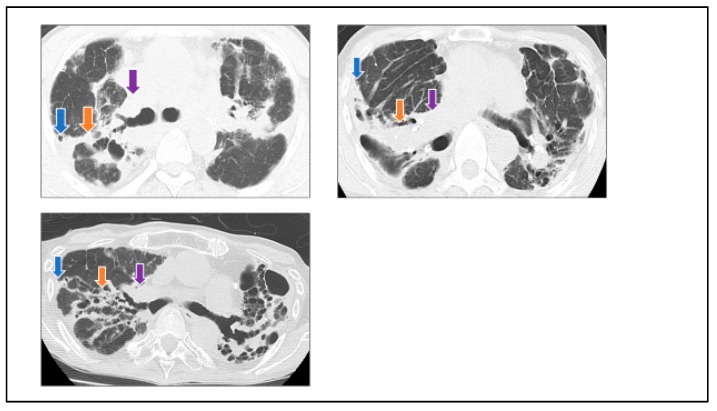
‘Central-peripheral band’. In this study, we defined areas of consolidations around the bronchovascular bundle that progress towards the mediastinum (purple arrows: *central* consolidation) and pleura (blue arrows: *peripheral* consolidation) as ‘central-peripheral band’ (orange arrows).

CT findings were evaluated for multiple granular/nodular opacities distributed along the bronchovascular bundle; ground-glass opacities (GGOs), defined as areas of hazy increased attenuation with preservation of bronchial and vascular margins; consolidations, defined as areas of homogeneous increased attenuation that obscured the pulmonary vasculature; ‘central-peripheral band’ (Figure 1), defined as areas of consolidations that progressed around the bronchovascular bundle both centrally near the mediastinum and peripherally right under the pleura; traction bronchiectasis, defined as irregular bronchial dilatation within or around areas with parenchymal abnormality; honeycomb lung-like structures [1], defined as areas of cystic spaces with thickened walls right under the pleura; cysts including bullae; lobe shrinkage; lymphadenopathy, defined as lymph nodes with a ≥1 cm short-axis diameter; calcification of lymph nodes; and pulmonary artery dilatation, defined as the ratio of the main pulmonary artery to the ascending aorta ≥1. Spatial distributions of abnormalities were designated as *central*, defined as the region from around the trachea to the fifth bronchus, and *peripheral*, defined as the region from the sixth bronchus to the area right under the pleura.

In addition, lesion volume was estimated and graded in 5% increments from 0% (absent) to 100% (whole lung). The degrees of calcification of lymph nodes were semi-quantitatively graded as 0 (absent), 1 (mild), 2 (moderate), or 3 (severe).

For this study, a pulmonologist with 35 years’ experience and a radiologist with 30 years’ experience, who were unaware of the clinical information, retrospectively and independently evaluated the lung CT findings. Disagreements between them after their first assessment were resolved by discussion. The kappa coefficient was used to measure inter-observer reliability, with coefficients of <0.21, 0.21–0.40, 0.41–0.60, 0.61–0.80, and >0.80 considered to indicate poor, fair, moderate, good, and excellent agreement, respectively. Spearman’s rank correlation coefficient was also used to measure inter-rater reliability during the estimation of lesion volume and calcification of lymph nodes, with coefficients of <0.19, 0.2–0.39, 0.4–0.69, 0.7–0.89, and >0.90 considered to indicate a very weak, weak, moderate, strong, and very strong correlation, respectively. Findings of chest CT taken at the time of oxygen therapy induction were retrospectively evaluated. Disease progression of each CT finding during the time between the first CT and oxygen therapy induction was also retrospectively evaluated.

### 2.3. Data Analysis

Continuous data were expressed as median with interquartile range (IQR) for nonparametric data. Categorical data were presented as absolute numbers and relative frequencies (*n*, %). To calculate kappa coefficient and Spearman’s rank correlation coefficient, we used Statistical Package for the Social Sciences (SPSS Version 25).

## 3. Results

### 3.1. Patient Characteristics

The characteristics and clinical manifestations of the 10 study patients are summarized in Table 1. All 10 patients received home oxygen therapy. Median age (interquartile range (IQR)) at the time of oxygen therapy induction was 62.5 (50.5–67.75) years (Table 1). Four were former/current smokers. Median age (IQR) at the time of sarcoidosis onset was 30.0 (27.25–34.0) years, and sarcoidosis was diagnosed histologically in all patients although the site of biopsy was unknown in one patient. Median observation period between sarcoidosis onset and oxygen therapy induction (IQR) was 28 (15–34.5) years, and 8 of the 10 patients received systemic steroids. Median radiological observation period with chest CT between first CT and oxygen therapy induction (IQR) was 8 (5.5–10.25) years.

Before oxygen therapy induction, restrictive ventilatory impairment was found in eight patients (8/8). Wheezing developed during follow-up in five patients (5/10), and respiratory function tests detected obstructive ventilatory impairment in three of eight patients (3/8). Impaired diffusion capacity was found at least in four patients (4/6). Pulmonary hypertension (PH) was detected clinically in at least six patients by ultrasonic echocardiography.

### 3.2. Inter-Observer Reliability

The kappa coefficients and Spearman’s rank correlation coefficients are summarized in Table 2. Agreement between the raters was good in terms of assessments for these CT findings.

### 3.3. Radiological Findings at the Time of Oxygen Therapy Induction

#### 3.3.1. CT Findings

The CT findings of these patients at the time of oxygen therapy induction are summarized in Table 3. The upper lung field was mainly involved. The predominant CT findings were consolidations distributed along the bronchovascular bundles comprising ‘central-peripheral band’, traction bronchiectasis, peripheral cysts/bullae, shrinkage of upper lobe (SUL), and hilar mediastinal lymphadenopathy with calcification. 

In detail, consolidations were found in nine patients (average lesion volume 23.0%; central in eight patients, peripheral in seven patients), and ‘central-peripheral band’ in six patients. Traction bronchiectasis was found in eight patients (average lesion volume 24.5%: central in eight patients, peripheral in seven patients), and honeycomb lung-like structures in three patients (average lesion volume 5.5%). Cysts were seen in nine patients (average lesion volume 30.0%; central in zero patients, peripheral in nine patients). Shrinkage of the upper lobes was seen in seven patients, among whom two patients also had lower lobe shrinkage. Multiple granular/nodular opacities distributed along the bronchovascular bundles were found in one patient (average lesion volume 5.0%; central in one patient, peripheral in one patient), and GGO in four patients (average lesion volume 6.0%; central in zero patients, peripheral in four patients). Hilar mediastinal lymphadenopathy was found in eight patients, calcification of these lymph nodes in nine patients (mild calcification in two patients, moderate calcification in three patients, and severe calcification in four patients) and of lung lesions in one patient. Thoracic flattening was seen in one patient, and PH in five patients.

#### 3.3.2. Correlation between Each CT Finding

Consolidations consisting of ‘central-peripheral band’, traction bronchiectasis clusters, peripheral cysts, and upper lobe shrinkage seemed to correlate with each other. 

**Figure 2:**
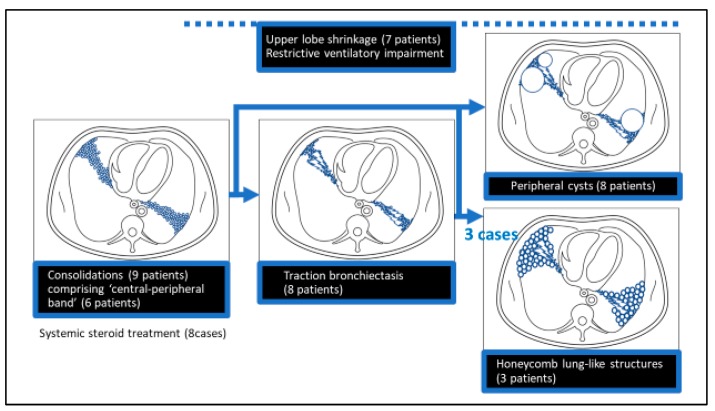
Postulated schema for the pathogenesis of peripheral cysts and honeycomb lung-like structures. Among the nine patients with consolidations, six patients had ‘central-peripheral band’, eight patients had traction bronchiectasis, eight patients had peripheral cysts, seven patients had upper lobe shrinkage, and three patients had honeycomb lung-like structures.

Traction bronchiectasis seemed to arise from granular/nodular opacities and consolidations distributed along the bronchovascular bundle, which are components of ‘central-peripheral band’. Clustering of traction bronchiectasis at the distal side formed honeycomb lung-like structures in three patients. Peripheral cysts were detected in nine patients, and at least three patients had cysts that connected with the distal side of traction bronchiectasis, while two patients without traction bronchiectasis also had severe multiple cyst formation. Among nine patients with consolidations, six patients had ‘central-peripheral band’; eight patients had traction bronchiectasis; eight patients had peripheral cysts; seven patients had upper lobe shrinkage; three patients had honeycomb lung-like structures (Figure 2).

Among the seven patients with upper lobe shrinkage, all patients had consolidations and traction bronchiectasis; six patients had ‘central-peripheral band’; six patients had peripheral cysts; and two patients had honeycomb lung-like structures. Three patients without upper lobe shrinkage included the two above-mentioned patients with severe multiple cysts without traction bronchiectasis and one patient with honeycomb lung-like structures.

### 3.4. Changes in Radiological Findings between the First CT and Oxygen Therapy Induction

#### 3.4.1. Progression of Consolidations Comprising ‘Central-Peripheral Band’, Traction Bronchiectasis Clusters, Peripheral Cysts, and Upper Lobe Shrinkage

The progression of fibrotic pulmonary sarcoidosis was characterized by the progression of consolidations, ‘central-peripheral band’, traction bronchiectasis, honeycomb lung-like structures, peripheral cysts, and upper lobe shrinkage. In detail, the average lesion volume of consolidations increased from 14.0% to 23.0% though the number of patients with this finding did not change (Table 3, Table 4). The number of patients with ‘central-peripheral band’ increased from five to six. The number of patients with traction bronchiectasis increased from six to eight (average lesion volume: from 11.0% to 24.5%). The number of patients with honeycomb lung-like structures increased from two to three (average lesion volume: from 1.0% to 5.5%). The number of patients with peripheral cysts increased from seven to nine (average lesion volume: from 18.0% to 30.0%). The number of patients with upper lobe shrinkage increased from six to seven, and patients with lower lobe shrinkage increased from zero to two.

Increased lesion volume was observed for consolidations together with traction bronchiectasis clusters and peripheral cysts. Among eight patients with increased lesion volume of consolidation during the time between the first CT and oxygen therapy induction, seven patients also showed increased lesion volume of traction bronchiectasis, five patients showed increased lesion volume of cysts, and two patients had increased lesion volume of honeycomb lung-like structures.

#### 3.4.2. Disappearance of Granular/Nodular Opacities and GGOs

Some of the granular/nodular opacities and GGOs, which are distributed along the bronchovascular bundle, disappeared during the chronic clinical course. The number of patients with multiple granular/nodular opacities decreased from two to one (average lesion volume from 8.0% to 5.0%). Though the number of patients with GGO did not change, the average lesion volume decreased from 12.5% to 6.0%.

#### 3.4.3. Progression of Calcification of Hilar and Mediastinal Lymph Nodes

The number of patients with moderate or severe calcification of hilar and mediastinal lymph nodes increased from three to seven, while that of patients with no or mild calcification of these lymph nodes decreased from seven to three.

### 3.5. Comorbidities and Prognosis after Oxygen Therapy Induction

Median observation period (IQR) after oxygen therapy induction was 3.5 (1.75–4.25) years. During this period, chronic progressive pulmonary aspergillosis (CPPA) was detected clinically in four patients, all of whom died during this observation period. Three patients developed pneumothorax (two of them had bilateral pneumothorax). 

In total, seven patients died during this period, six of whom progressed to type Ⅱ respiratory failure at the terminal stage. The main causes of death were chronic respiratory failure in two patients, CPPA in two patients, other respiratory infection in one patient, PH in one patient, and thalamic haemorrhage in one patient. 

## 4. Discussion

This study examined CT findings and observed disease progression in 10 consecutively extracted pulmonary sarcoidosis patients who developed chronic respiratory failure. The median observation period (IQR) between sarcoidosis onset and oxygen therapy induction was 28 (15–34.5) years. There are five important observations from this study. First, during the progression of pulmonary fibrosis and respiratory failure, consolidations comprising ‘central-peripheral band’ (Figure 1), traction bronchiectasis clusters, peripheral cysts/bullae, upper lobe shrinkage, and calcification of hilar and mediastinal lymph nodes progressed with disappearance of some of the granular/nodular opacities and GGOs, which are distributed along the bronchovascular bundles. Second, traction bronchiectasis seemed to arise from these granular/nodular opacities and consolidations comprising ‘central-peripheral band’. Clustering of traction bronchiectasis at the distal side formed honeycomb lung-like structures in three patients. At the time of oxygen therapy induction, among nine patients with consolidations, six patients had ‘central-peripheral band’; eight patients had traction bronchiectasis; eight patients had peripheral cysts; seven patients had upper lobe shrinkage; and three patients had honeycomb lung-like structures (Figure 2). Third, upper lobe shrinkage (seven patients) progressed together with progression of consolidations (seven patients) comprising ‘central-peripheral band’ (six patients), traction bronchiectasis (seven patients), honeycomb lung-like structures (two patients), and peripheral cysts (six patients). In contrast, three patients without upper lobe shrinkage included two patients with severe multiple cyst formation without traction bronchiectasis, as well as one patient with honeycomb lung-like structures. Fourth, restrictive ventilatory impairment developed in most patients as fibrosis progressed, in addition to obstructive ventilatory impairment and impaired diffusion capacity. Finally, PH was detected radiologically in five patients, CPPA in four patients, and pneumothorax in three patients. During the median observation period (IQR) after oxygen therapy induction of 3.5 (1.75–4.25) years, seven patients died.

Given that sarcoidosis is a relatively rare disease and decades of observation are required before development of pulmonary fibrosis is seen, it is difficult to find multiple patients with this condition. Among more than 2500 outpatients and inpatients treated at participating hospitals, we found only 10 patients with this condition.

When considering the first observation made in this study, areas of consolidations around the bronchovascular bundle that progressed towards the mediastinum and the pleura were defined as ‘central-peripheral band’ (Figure 1), and this may reflect the lymphatic flow through which inhaled antigen-loaded antigen-presenting cells move. In the lung, lymph flows toward the hilum in the direction of the pulmonary artery and vein and bronchi as well as around the pleura; flow directly into the mediastinal lymph nodes from the visceral pleura to the mediastinal pleura has also been confirmed [7].

Concerning the second observation, the honeycomb lung-like structures seemed to be the result of clustering of traction bronchiectasis and small cysts at the distal side (Figure 2). To our knowledge, this finding has not been reported to date, partly because the number of reports that investigated such patients are limited [8,9,10,11,12,13,14]. However, considering the relatively low incidence of honeycomb lung-like structures (30%) in this study, there remains a possibility that this architecture does not play a crucial role in disease prognosis.

Regarding the third observation, consolidations comprising ‘central-peripheral band’ and traction bronchiectasis clusters may play important roles in the mechanism of upper lobe shrinkage, a finding that has been widely known. Indeed, bronchovascular bundle fibrosis was frequently observed (58%) with accompanying peribronchial atelectasis in a pathological study of 66 autopsy cases [15]. However, considering the two cases of severe multiple cyst formation without upper lobe shrinkage or traction bronchiectasis, both of whom developed wheezing and had PH and CPPA in common, a morphological sub-classification of fibrotic pulmonary sarcoidosis may be necessary based on how it develops, especially with/without upper lobe shrinkage and traction bronchiectasis (Figure 2).

The mechanisms of peripheral cyst formation after disappearance of granular/nodular opacities and consolidations, distributed along the bronchovascular bundles, may include stenosis of bronchi with granulomatous involvement and peribronchial fibrosis, and further check-valve mechanism of bronchiolar involvement of granulomata and fibrosis [15]. In this study, some cysts were connected with the distal side of traction bronchiectasis, and temporal size reduction of enlarged cysts was sometimes observed, suggesting the possibility of these factors. In contrast, we also noted the formation of severe multiple cysts with lesion volumes that occupied more than half of the total lung in the two above-mentioned patients, suggesting the possibility of other factors. An experimental study reported that impaired lymphatic flow in mice resulted in hypoxia and features of lung injury resembling emphysema in association with a pulmonary inflammatory state characterized by formation of tertiary lymphoid organs [16].

On the fourth observation, obstructive ventilatory impairment has been of interest in pulmonary sarcoidosis, but this study showed that restrictive ventilatory impairment develops in most patients as fibrosis progressed. Investigation of each CT finding in relation to each type of pulmonary dysfunction is an important task for future studies.

Regarding the final observation, in the progression of fibrosis, accompanying PH is considered to contribute to chronic respiratory failure. PH in sarcoidosis is classified as Group 5 (PH with multifactorial mechanisms) in the clinical classification of PH updated at the recent world symposium on PH (Nice, France, 2013) [17], because extrapulmonary lesions (e.g., cardiac, vascular, and hepatic lesions), in addition to parenchymal lung damage and capillary obstruction due to fibrosis, are involved in the development of PH. CPPA was suggested to be as important as PH in the vital prognosis of pulmonary fibrosis in this study.

The main strength of our study is the long median observation period (IQR) between sarcoidosis onset and oxygen therapy induction of 28 (15–34.5) years. However, the study has some limitations, including potential selection bias because of the limited number of patients, all of whom visited the respiratory center of three hospitals. Therefore, the present results might not be generalizable to sarcoidosis patients as a whole. Furthermore, because we examined past medical records, the possible information bias could reduce the validity of the study. 

In conclusion, during the progression of fibrotic pulmonary sarcoidosis, consolidations comprising ‘central-peripheral band’ progressed together with traction bronchiectasis clusters and peripheral cysts, which results in upper lobe shrinkage. Honeycomb lung-like structures seemed to be the result of clustering of traction bronchiectasis at the distal side. Restrictive ventilatory impairment developed in most patients as fibrosis progressed. This may lead to respiratory failure with possible complications such as PH and CPPA.

## Figures and Tables

**Table 1:** Patient characteristics.

Case No.	1	2	3	4	5	6	7	8	9	10	Total (*n*)
Age at the time of oxygen therapy induction	58	37	40	54	66	60	65	70	78	67	
Female	+				+	+	+	+	+	+	7
Former/current smoker		+	+						+	+	4
(pack-years)		(*)	(14.0)						(16.5)	(10.0)	
Site of biopsy	*	Skin, liver	Lung	Lung	Lung	Lung	Skin	Scalene lymph node	Lung, skin	Skin	
Organ involvement											
Lungs ^#^	+	+	+	+	+	+	+	+	+	+	10
Eye				+				+	+	+	4
Skin		+					+		+	+	4
Liver		+									1
Scalene lymph node								+			1
Gingiva										+	1
Hematopoietic organ					+						1
Treatment for sarcoidosis											
ICS	+		+		+	+			+		5
PSL/mPSL		+	+	+	+	+		+	+	+	8
MTX			+						+	+	3
Lung function											
Restrictive ventilatory impairment	+	+	+	+	+	+	+	+	*	*	
Obstructive ventilatory impairment		+	+		+				*	*	
Impaired diffusion capacity	*	+	*		*	+	+		+	*	
Interval between sarcoidosis onset and oxygen therapy induction	28 years	7 years	12 years	24 years	32 years	28 years	31 years	49 years	16 years	42 years	

+, yes; blank, no; * not available; ^#^ including bilateral hilar-mediastinal lymphadenopathy (BHL). ICS, inhaled corticosteroid; PSL, prednisolone; mPSL, methylprednisolone; MTX, methotrexate.

**Table 2:** Inter-class reliability.

Findings	Kappa Coefficient	Spearman’s Rank Correlation Coefficient
**Parenchyma**		
Multiple granular/nodular opacity along the lymphatic tracts (%)	1.000	1.000
Ground-glass opacity (%)	1.000	0.996
Consolidation (%)	0.950	0.985
Central-peripheral band	1.000	
Traction bronchiectasis (%)	1.000	0.998
Honeycomb lung-like structures (%)	1.000	1.000
Cysts (%)	1.000	0.990
Lobe shrinkage	1.000	
Mediastinum		
Hilar Mediastinal lymphadenopathy	1.000	
Calcification of lymph node	1.000	1.000
Pulmonary artery dilatation	1.000	
Distribution		
Central	1.000	
Peripheral	0.983	

**Table 3:** CT findings at the time of oxygen therapy induction (*n* = 10).

Findings	1	2	3	4	5	6	7	8	9	10	Total (*n*)
**Parenchyma**											
Multiple granular/nodular opacities (%)	0	0	0	0	0	0	0	0	0	50	
Central										+	1
Peripheral										+	1
Ground-glass opacity (%)	0	0	20	10	10	20	0	0	0	0	
Central											0
Peripheral			+	+	+	+					4
Consolidation (%)	25	0	5	30	15	25	40	35	15	40	
Central	+		+	+		+	+	+	+	+	8
Peripheral	+			+	+	+	+	+		+	7
Central-peripheral band	+			+		+	+	+		+	6
Traction bronchiectasis (%)	20	0	0	20	35	45	40	30	50	5	
Central	+			+	+	+	+	+	+	+	8
Peripheral	+			+	+	+	+	+	+		7
Honeycomb lung-like structures (%)	0	0	0	10	5	0	0	0	40	0	
Cysts (%)	10	80	65	10	50	10	30	35	10	0	
Central											0
Peripheral	+	+	+	+	+	+	+	+	+		9
Lobe shrinkage											
Upper lobe	+			+	+	+	+	+		+	7
Lower lobe				+						+	2
**Mediastinum**											
Hilar mediastinal lymphadenopathy		+	+	+	+		+	+	+	+	8
Calcification of lymph nodes											
No		+									1
Mild						+			+		2
Moderate	+			+	+						3
Severe			+				+	+		+	4
Pulmonary artery dilatation	+	+	+		+	+					5

+, yes; blank, no.

**Table 4:** CT findings at the time of first CT (*n* = 10).

Findings	1	2	3	4	5	6	7	8	9	10	Total (*n*)
**Parenchyma**											
Multiple granular/nodular opacity (%)	0	0	0	0	0	0	40	0	0	40	
Central							+			+	2
Peripheral							+			+	2
Ground-glass opacity (%)	0	0	30	5	0	50	0	0	40	0	
Central											0
Peripheral			+	+		+			+		4
Consolidation (%)	20	0	10	25	10	20	20	10	5	20	
Central	+		+	+		+	+		+		6
Peripheral	+		+	+	+	+	+	+		+	8
Central-peripheral band	+			+		+	+	+			5
Traction bronchiectasis (%)	20	0	0	15	30	25	0	10	10	0	
Central	+			+	+	+		+	+		6
Peripheral	+			+	+	+		+	+		6
Honeycomb lung-like structures (%)	0	0	0	0	5	0	0	0	5	0	
Cysts (%)	10	60	60	0	30	0	5	5	10	0	
Central											0
Peripheral	+	+	+		+		+	+	+		7
Lobe shrinkage											
Upper lobe	+			+	+	+	+	+			6
Lower lobe											0
**Mediastinum**											
Hilar mediastinal lymphadenopathy		+	+	+	+		+	+	+	+	8
Calcification of lymph node											
No		+									1
Mild	+			+	+	+	+		+		6
Moderate			+					+			2
Severe										+	1
Pulmonary artery dilatation	+	+	+		+	+					5

+, yes; blank, no.

## Abbreviations

BHL: bilateral hilar-mediastinal lymphadenopathy; CT: computed tomography; GGO: Ground-grass opacity; IQR: interquartile range; PH: pulmonary hypertension; CPPA: chronic progressive pulmonary aspergillosis.
